# Evaluation of the Usefulness of CO-RADS for Chest CT in Patients Suspected of Having COVID-19

**DOI:** 10.3390/diagnostics10090608

**Published:** 2020-08-19

**Authors:** Tomoyuki Fujioka, Marie Takahashi, Mio Mori, Junichi Tsuchiya, Emi Yamaga, Toshihiro Horii, Hirofumi Yamada, Mizuki Kimura, Koichiro Kimura, Yoshio Kitazume, Mitsuhiro Kishino, Ukihide Tateishi

**Affiliations:** Department of Diagnostic Radiology, Tokyo Medical and Dental University, Tokyo 113-8510, Japan; tkhsdrnm@tmd.ac.jp (M.T.); m_mori_116@yahoo.co.jp (M.M.); tcymrad@tmd.ac.jp (J.T.); ymgdrnm@tmd.ac.jp (E.Y.); horii.m66.tmdu@gmail.com (T.H.); hrfmymd1994@gmail.com (H.Y.); 150421ms@tmd.ac.jp (M.K.); kmrdrnm@tmd.ac.jp (K.K.); ktzmmrad@tmd.ac.jp (Y.K.); ksnmrad@tmd.ac.jp (M.K.); ttisdrnm@tmd.ac.jp (U.T.)

**Keywords:** chest imaging, computed tomography, COVID-19, CO-RADS, radiology, pneumonia

## Abstract

The purpose of this study was to use the Coronavirus Disease 2019 (COVID-19) Reporting and Data System (CO-RADS) to evaluate the chest computed tomography (CT) images of patients suspected of having COVID-19, and to investigate its diagnostic performance and interobserver agreement. The Dutch Radiological Society developed CO-RADS as a diagnostic indicator for assessing suspicion of lung involvement of COVID-19 on a scale of 1 (very low) to 5 (very high). We investigated retrospectively 154 adult patients with clinically suspected COVID-19, between April and June 2020, who underwent chest CT and reverse transcription-polymerase chain reaction (RT-PCR). The patients’ average age was 61.3 years (range, 21–93), 101 were male, and 76 were RT-PCR positive. Using CO-RADS, four radiologists evaluated the chest CT images. Sensitivity, specificity, and area under the receiver operating characteristic curve (AUC) were calculated. Interobserver agreement was calculated using the intraclass correlation coefficient (ICC) by comparing the individual reader’s score to the median of the remaining three radiologists. The average sensitivity was 87.8% (range, 80.2–93.4%), specificity was 66.4% (range, 51.3–84.5%), and AUC was 0.859 (range, 0.847–0.881); there was no significant difference between the readers (*p* > 0.200). In 325 (52.8%) of 616 observations, there was absolute agreement among observers. The average ICC of readers was 0.840 (range, 0.800–0.874; *p* < 0.001). CO-RADS is a categorical taxonomic evaluation scheme for COVID-19 pneumonia, using chest CT images, that provides outstanding performance and from substantial to almost perfect interobserver agreement for predicting COVID-19.

## 1. Introduction

In the city of Wuhan, Hubei province, China, a highly contagious disease was first identified at the end of 2019, which was later proven to be caused by a novel coronavirus (2019-nCoV or severe acute respiratory syndrome coronavirus 2) [1]. As of 10 August 2020, more than 19,718,000 people worldwide have been infected with coronavirus disease 2019 (COVID-19), and more than 728,000 have died [2].

Early diagnosis of COVID-19 is crucial for the isolation of patients and prevention of the spread of infection, as well as early patient intervention. The gold standard for COVID-19 diagnosis is the reverse transcription-polymerase chain reaction (RT-PCR) assay. However, its sensitivity is variable as it depends on the duration of symptoms, viral load, the rate of viral replication in the upper respiratory tract, and quality of the test sample, and has a reported pooled sensitivity of 64.8% (95% confidence interval (CI) 54.5–74.0) in clinical practice [3].

Computed tomography (CT) examination plays an important role in epidemic areas in the diagnosis of COVID-19 because of its high sensitivity [4,5]. It has been reported that patients with negative RT-PCR results may have positive chest CT findings, and combining RT-PCR with CT scans is expected to improve the diagnosis of COVID-19 [4,5]. Several CT image findings characteristic of COVID-19 pneumonia have already been reported, the most common of which are bilateral, peripheral/subpleural, or posterior ground-glass opacities with or without consolidations [6,7,8,9].

The Dutch Radiological Society created a COVID-19 Reporting and Data System (CO-RADS) that standardizes the assessment scheme and simplifies reporting with a five-point scale of suspicion for COVID-19 pneumonia in chest CT images [8]. The diagnostic performance of CO-RADS for COVID-19 through the interpretation of chest CT images was shown to be excellent, and the interobserver agreement was moderate to substantial [8].

It is known that the incidence, severity, and mortality of COVID-19 pneumonia vary between races and countries [10]; therefore, it is necessary to verify whether the CO-RADS findings from Europe can be applied to Japan. Therefore, we investigated the usefulness of CO-RADS using Japanese chest CT data.

## 2. Material and Methods

### 2.1. Patients

The Tokyo Medical and Dental University Hospital Ethics Committee approved this retrospective study (approval ID: M2020-054, approval date: 15 June 2020) and waived the requirement for written informed consent.

The inclusion criteria for enrolling patients were: (a) those who were suspected by a clinician of having COVID-19 based on symptoms and history of exposure; (b) those who underwent chest CT at our hospital or outside institutions from January 2020 to June 2020; and (c) those who were diagnosed as positive or negative for COVID-19 by one or more RT-PCR tests. The following patients were excluded: (1) those who were diagnosed with COVID-19 but whose duration of infection was unknown; and (2) those who were younger than 20 years of age. A board-certified radiologist with 10 years of imaging experience and a medical student reviewed the database of radiology reports and medical records at our institute, and they extracted chest CT images and clinical data (age, sex, comorbidities, time of onset, symptoms, and final diagnosis). The definitive diagnosis of the patients was made by an experienced clinician of our hospital based on chest X-ray, chest CT, laboratory findings, and clinical data in the follow-up. COVID-19-positive (*n* = 76) and COVID-19-negative (*n* = 78) patients were selected for this study.

### 2.2. Chest Computed Tomography (CT) Imaging

All patients underwent CT, but the protocols were variable. In total, 120 CT examinations were performed at our institution using the SOMATOM Edge Plus (Siemens Healthcare GmbH, Erlangen, Germany) with 64-detector rows, while 34 CT examinations were performed at outside institutions using several CTs with from 4 to 320-detector rows. The acquisition parameters at our hospital were as follows: 120 kV tube voltage with automatic tube current modulation (150 mAs); tube rotation time 0.28 s; beam collimation 128 ch × 0.6 mm; and beam pitch 1.5. By default, 2.0 mm without interslice gap chest CT images were reconstructed using sharp tissue kernel (Bl57) with the filtered back-projection technique. Slice thickness of reconstruction images ranged from 1.25 to 5 mm at outside institutions.

### 2.3. CO-RADS, the Coronavirus Disease 2019 (COVID-19) Reporting and Data System

CO-RADS assesses the level of suspicion for COVID-19 in lung lesions based on the features of chest CT findings. The degree of suspicion is classified into five levels from very low (CO-RADS 1) to very high (CO-RADS 5). Two additional categories encode technically inadequate tests (CO-RADS 0) and RT-PCR-proven COVID-19 infections (CO-RADS 6), at the time of the test, respectively (Table 1).

The CO-RADS categories are summarized as follows [8]:

1. CO-RADS 0 is chosen if none of the five categories can be assigned because of incomplete scans or poor quality.
2. CO-RADS 1 means a very low level of suspicion for pulmonary lesion resulting from COVID-19, based on either a normal CT or CT findings of unequivocal non-infectious etiology. Mild or severe emphysema, perifissural nodules, lung tumors, or fibrosis characterize CO-RADS 1.
3. CO-RADS 2 means a low level of suspicion for pulmonary lesion resulting from COVID-19, based on CT findings in the lungs typical of etiology indicative of infections other than COVID-19, including bronchitis, infectious bronchiolitis, bronchopneumonia, lobar pneumonia, and pulmonary abscess. Findings include a tree-in-bud sign, a centrilobular nodular pattern, lobar or segmental consolidation, and lung cavitation.
4. CO-RADS 3 means CT findings equivocal for pulmonary involvement of COVID-19 that can also be seen in other types of viral pneumonia or non-infectious etiologies. Findings include perihilar ground-glass, homogenous extensive ground-glass with or without sparing of some secondary pulmonary lobules, or ground-glass together with smooth interlobular septal thickening with or without pleural effusion in the absence of other typical CT findings.
5. CO-RADS 4 means a high level of suspicion for pulmonary lesion resulting from COVID-19, based on typical CT findings but exhibiting some overlap with other types of (viral) pneumonia. Findings are not located in contact with the visceral pleura or are located strictly unilaterally, have a predominantly peribronchial distribution, or overlap with severe diffuse preexisting pulmonary abnormalities.
6. CO-RADS 5 means a very high level of suspicion for pulmonary lesion resulting from COVID-19, based on typical CT findings. Obligatory features are ground-glass opacities, with or without consolidations, close to visceral pleural surfaces, including the fissures, and a multifocal bilateral distribution.

### 2.4. Readout

Chest CT images were reviewed by four radiologists (Reader 1 with 1 year, Reader 2 with 6 years, Reader 3 with 7 years, and Reader 4 with 10 years of experience) using CO-RADS. Reader 1 and Reader 2 were board-certified radiologists, and the remaining two were radiology residents. They evaluated the images using the medical viewing system EV Insite R (PSP Co., Tokyo, Japan), with feature reading tools.

Although they knew the age and sex of the patients, they were blind to other clinical information. Readers categorized each patient as CO-RADS 0, 1, 2, 3, 4, or 5.

### 2.5. Statistical Analysis

All statistical analyses in this study were performed using SPSS for Windows version 22.0 (IBM Corp., New York, NY, USA).

We performed a Student’s *t*-test or Chi-squared test to compare characteristics (age, sex, comorbidities) of patients with or without COVID-19. The sensitivity and specificity for differentiating COVID-19-positive from COVID-19-negative patients. Receiver operating characteristic (ROC) analyses were performed to calculate the area under the ROC curve (AUC) for evaluations of the four readers in distinguishing COVID-19-positive from COVID-19-negative patients using CO-RADS categorization. Then, the sensitivity and specificity were calculated using cut-off value ≥ Category 3. Interobserver agreement was assessed using the intraclass correlation coefficient (ICC). ICCs were calculated by comparing the CO-RADS score of each reader with the median of the remaining three readers and were interpreted as follows: <0.20, slight; 0.21–0.40, fair; 0.41–0.60, moderate; 0.61–0.80, substantial; and 0.81–1.0, almost perfect [11]. We considered a *p*-value of less than 0.05 to be statistically significant.

## 3. Results

Table 2 shows the characteristics of the 154 patients included in this study. The patients ranged in age from 21 to 93 years (mean ± SD; 61.3 ± 18.8), with 101 males and 53 females. All patients were clinically suspected of having COVID-19 and underwent one or more RT-PCR tests. Seventy-six of them tested positive with RT-PCR and were diagnosed with COVID-19. Among the COVID-19 patients, clinical presentations included fever (*n* = 67; 88.1%), cough (*n* = 32; 42.1%), dyspnea (*n* = 26; 34.2%), and fatigue (*n* = 13; 17.1%). There were no asymptomatic COVID-19 patients in this study. The time between symptom onset and the CT scan was between 0 and 27 days (mean ± SD; 8.6 ± 5.6).

Among the 78 COVID-19-negative patients, 36 were diagnosed as having pneumonia other than COVID-19, which were community-acquired pneumonia (*n* = 29), interstitial pneumonia (*n* = 4), drug-induced pneumonia (*n* = 1), eosinophilic pneumonia (*n* = 1), and lung abscess (*n* = 1). Fifteen patients were diagnosed with diseases other than pneumonia, which were pulmonary edema (*n* = 5), atelectasis (*n* = 4), lung tumor (*n* = 3), pleuritis (*n* = 2), and emphysema (*n* = 1). Twenty-seven patients were diagnosed as normal (Table 3). Patients without COVID-19 were older and had more comorbidities of cancer and collagen disease than the patients with COVID-19 (*p* = 0.006, <0.001 and 0.022). However, there was no significant difference between the two groups for sex ratio, comorbidity of cardiovascular disease, lung disease, and diabetes.

**Figure 1:**
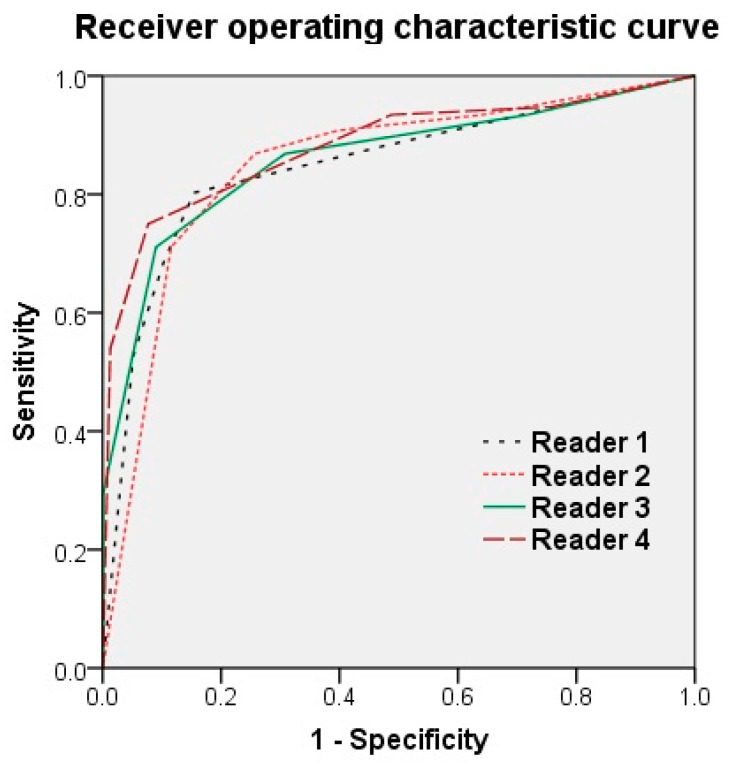
Receiver operating characteristic curve of the four readers. Reader 1, Reader 2, Reader 3, and Reader 4 had an area under the ROC curve (AUC) of 0.847, 0.849, 0.859, and 0.881, respectively. Although the AUC tended to increase with years of radiology experience, there was no significant difference between the readers (*p* > 0.200).

No cases were evaluated as CO-RADS Category 0. Reader 1, Reader 2, Reader 3, and Reader 4 had sensitivities, respectively, of 80.3%, 90.8%, 86.8%, and 93.4%, specificities of 84.6%, 60.3%, 69.2%, and 80.0% (cut off value ≥ Category 3), and AUCs of 0.847, 0.849, 0.859, and 0.881. Although the AUCs tended to increase with years of radiology experience, there was no significant difference between the readers (*p* > 0.200; Table 4; Figure 1).

**Figure 2:**
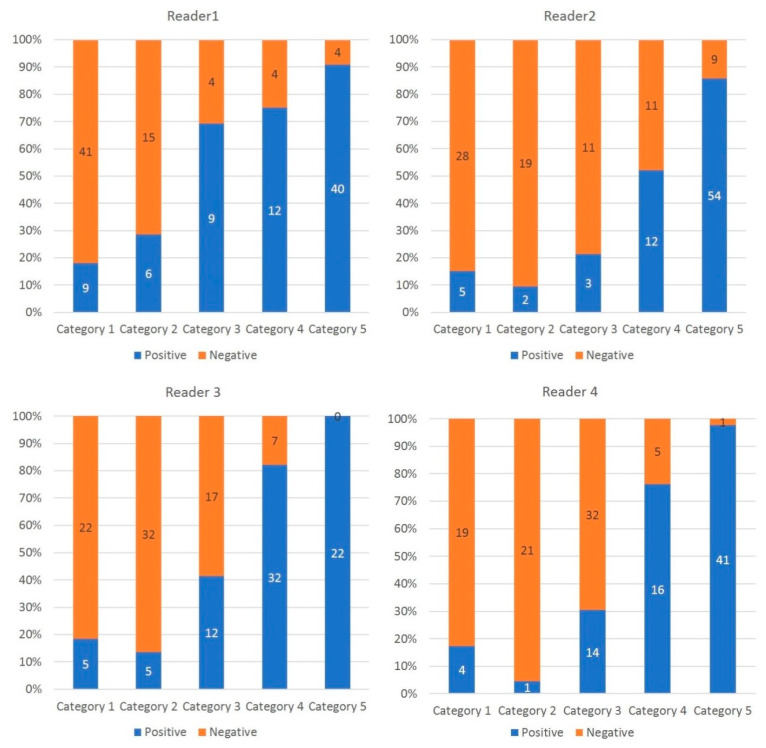
Percentage and number of COVID19 patients per CO-RADS category. The histogram shows the proportion of COVID19 patients per CO-RADS category. The higher the CO-RADS category, the higher the proportion of COVID-19 positive patients.

In 325 (52.8%) of all 616 observations, there was absolute agreement in the assignment CO-RADS category. A discrepancy of one CO-RADS category was seen in 214 (34.7%) of all 616, of two CO-RADS categories in 67 (3.7%) of all 616 and of three categories in 10 (0.1%) of all 616 observations (Figure 2).

Although the interobserver agreement for predicting COVID-19 of Reader 1 was substantial, that of the remaining three readers was almost perfect. The ICC average of readers was 0.840 (range, 0.800–0.874; Table 4).

**Figure 3:**
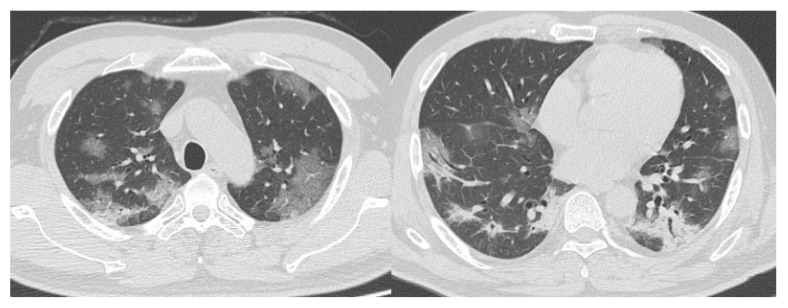
Representative computed tomography (CT) images of COVID-19 pneumonia (Case 1). Fifty-two-year-old man. The CT images show multifocal bilateral, peripheral/subpleural ground-glass opacities with consolidations. RT-PCR was positive for SARS-CoV-2. All readers diagnosed it as Category 5. COVID-19, coronavirus disease 2019; CT, computed tomography; RT-PCR, reverse transcription-polymerase chain reaction; SARS-CoV-2, severe acute respiratory syndrome coronavirus 2.

**Figure 4:**
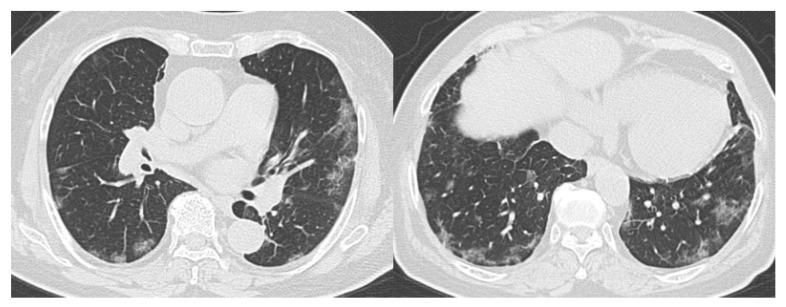
Representative CT images of COVID-19 pneumonia (Case 2). Seventy-seven-year-old female. The CT images show multifocal bilateral, peripheral/subpleural ground-glass opacities with curvilinear bands, and subpleural sparing. RT-PCR was positive for SARS-CoV-2. All readers diagnosed it as Category 5. COVID-19, coronavirus disease 2019; CT, computed tomography; RT-PCR, reverse transcription-polymerase chain reaction; SARS-CoV-2, severe acute respiratory syndrome coronavirus 2.

**Figure 5:**
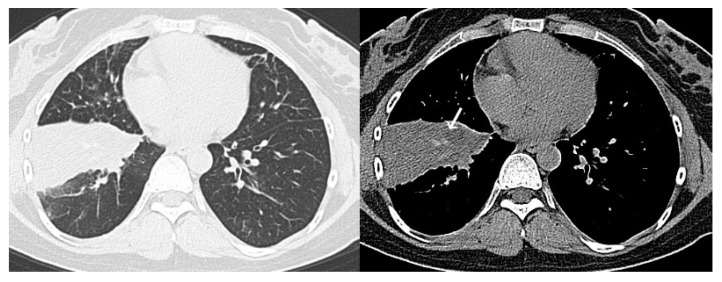
Representative CT images of a COVID-19-negative patient. Thirty-four-year-old female. The CT images show segmental consolidation with mucus plug (arrow). RT-PCR was negative for SARS-CoV-2 and she was diagnosed with community-acquired pneumonia. All readers diagnosed it as Category 2. COVID-19, coronavirus disease 2019; CT, computed tomography; RT-PCR, reverse transcription-polymerase chain reaction; SARS-CoV-2, severe acute respiratory syndrome coronavirus 2.

**Figure 6:**
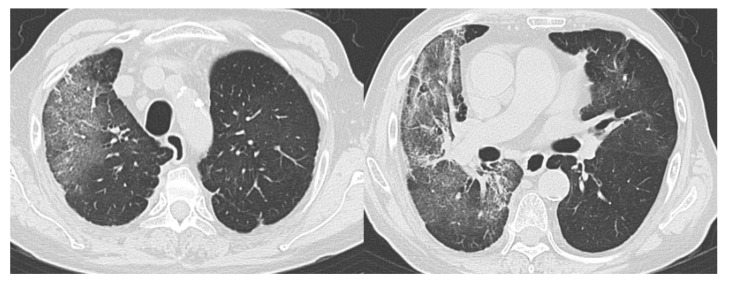
Example of a false-positive case (Case 1). Seventy-four-year-old male. The CT images show bilateral ground-glass opacities distributed peripherally to centrally with a right lung predominance. RT-PCR was negative for SARS-CoV-2. She had a history of eosinophilic pneumonia and was diagnosed with relapse of eosinophilic pneumonia. Readers 1, 2, 3, and 4 diagnosed it as Categories 3, 4, 4, and 3, respectively. CT, computed tomography; RT-PCR, reverse transcription-polymerase chain reaction; SARS-CoV-2, severe acute respiratory syndrome coronavirus 2.

**Figure 7:**
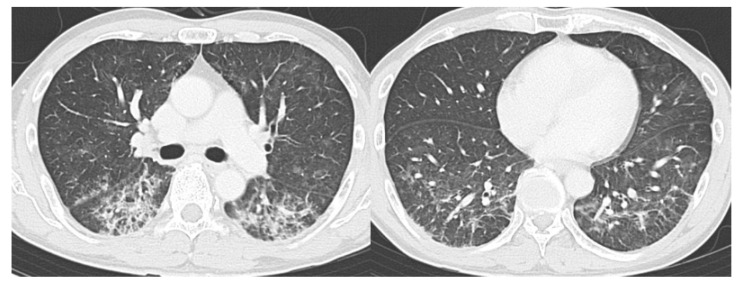
Example of a false-positive case (Case 2). Fifty-four-year-old female. The CT images show bilateral, peripheral posterior ground-glass opacities with consolidation with a lower lobe predominance. RT-PCR was negative for SARS-CoV-2. She had a history of medication use and was diagnosed with drug-induced pneumonia. Readers 1, 2, 3, and 4 diagnosed it as Categories 5, 4, 4, and 3, respectively. CT, computed tomography; RT-PCR, reverse transcription-polymerase chain reaction; SARS-CoV-2, severe acute respiratory syndrome coronavirus 2.

**Figure 8:**
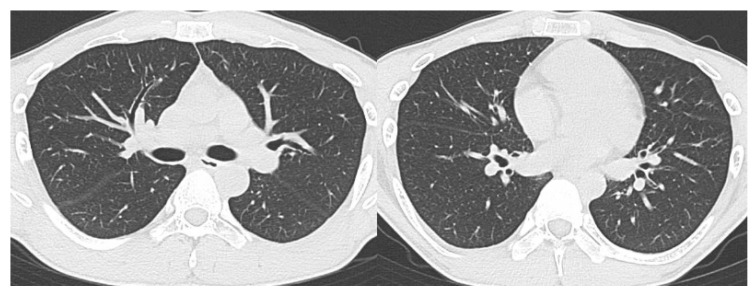
Example of a false-negative case. Twenty-five-year-old male. Although RT-PCR was positive for SARS-CoV-2, the CT images show no abnormal findings. All readers diagnosed it as Category 1. CT, computed tomography; RT-PCR, reverse transcription-polymerase chain reaction; SARS-CoV-2, severe acute respiratory syndrome coronavirus 2.

Figure 3; Figure 4 show representative true-positive cases, and Figure 5 represents a true negative case. Table 5; Table 6 list the CO-RADS category and characteristics of the cases that were diagnosed as false positives (i.e., non-COVID-19 patients with the median of Category 4 or 5) or false negatives (i.e., COVID-19 patients with the median of Category 1 or 2 by readers). There were 10 false-positive and 6 false-negative cases. The final diagnoses of the false-positive cases were community-acquired pneumonia (*n* = 5), interstitial pneumonia (*n* = 1), drug-induced pneumonia (*n* = 1), eosinophilic pneumonia (*n* = 1), pulmonary edema (*n* = 1), and pleuritis. Many of the false-negative cases were young patients. One patient was in his 90s and had no comorbidities. The other five patients were in their 20 s or 30 s. Figure 6; Figure 7 show representative false-positive cases, and Figure 8 represents a false-negative case.

## 4. Discussion

COVID-19 is an alarming infectious disease that is raging around the world. COVID-19 pneumonia, as has been reported, can cause severe respiratory failure and even death, especially in the elderly and patients with comorbidities [12,13].

The CO-RADS classification devised by the Dutch Radiological Society is a simple interpretation method with a high inspection accuracy and high matching rate. The widespread adoption of CO-RADS is expected to enable standardization of CT image interpretation worldwide but evidence for it is still insufficient. This is the first study, to our knowledge, to verify the effectiveness of CO-RADS with Japanese data.

The diagnostic performance of CT for COVID-19 had an average sensitivity of 87.8%, specificity of 66.4%, and AUC of 0.859. Although radiologists with varying years of experience assessed the images, there was no significant difference between the readers (*p* > 0.200). Moreover, there was substantial to almost perfect interobserver agreement among the readers. The average ICC of readers was 0.840 (range, 0.800–0.874).

It has been reported that in epidemic areas CT scans play an important role in diagnosing COVID-19 because of their high sensitivity, and combining RT-PCR with CT scans is expected to improve the diagnosis of COVID-19 [4,5]. This study was conducted in a population where approximately half of the patients were COVID-19 positive and all COVID-19 positive patients were symptomatic, and we were able to achieve high diagnostic performance results using CT scans. However, in areas with lower prevalence, it has been reported that their specificity and positive predictive value are lower. Further validation is needed as to whether CO-RADS is useful for diagnosing COVID-19 in non-endemic areas [14]. It is also known that young people without comorbidities are often asymptomatic or mildly symptomatic, even if infected with COVID-19 [15].

Despite the patients in this study being suspected of having pneumonia because of their symptoms and, thereby, being diagnosed with COVID-19, 6/76 (7.9%) patients had no CT findings of pneumonia. One patient was an older man with no comorbidities, and the other five patients were in their 20 s or 30 s. Because there is insufficient evidence for the effectiveness of screening patients who are asymptomatic or have mild symptoms, especially if they are young, it is necessary to thoroughly verify whether CT scans should be used for such patients.

This study also included 10 false-positive cases, consisting of community-acquired pneumonia, interstitial pneumonia, drug-induced pneumonia, eosinophilic pneumonia, pulmonary edema, and pleuritis. Because the image findings of these diseases overlap with that of COVID-19 pneumonia, it was difficult to make a perfect differential diagnosis, even with CO-RADS. When diagnosing patients suspected of having COVID-19 pneumonia, it is crucial to make a comprehensive assessment based on both CT images and clinical findings. In recent years, artificial intelligence, especially deep learning, has been greatly developed and applied to medical imaging [16,17,18,19]. Ni et al. utilized a deep learning model for automatic detection of abnormalities in chest CT images of COVID-19 patients. They demonstrated the deep-learning model performed exceptionally in detecting COVID-19 pneumonia on chest CT and could assist radiologists in making quicker diagnoses with superior diagnostic performance [20]. Further research will be needed to compare the diagnostic performance of deep learning algorithms with human readers, and to verify whether deep learning can assist radiologists and improve their accuracy and efficiency in the diagnosis of COVID-19.

This study had several limitations. First, our retrospective study was conducted at a single institution. Thus, to validate the findings of this research, more extensive, multicenter studies are necessary. Second, we used several CT systems from different companies. This may have caused variations in image quality, which in turn may have affected the diagnostic performance results of our readers. Third, the diagnostic criteria for COVID-19 in this study were defined as the results of RT-PCR, but it is known that these diagnoses are not perfect. Some patients may have false negatives or false positive Finally, we did not examine the relationship between CT image findings and severity and prognosis of disease [21]. Moreover, the timing of execution of chest CT scan after the onset of symptoms was inhomogeneous, and this fact represents a limitation, as chest abnormalities change during the diseases. Future research is necessary to clarify these relationships.

## 5. Conclusions

CO-RADS is a categorical taxonomic evaluation scheme for COVID-19 pneumonia, using chest CT images, 1 provides outstanding performance and from substantial to almost perfect interobserver agreement for predicting COVID-19. Interpretation of chest CT images using CO-RADS may contribute to the diagnosis and management of COVID-19 pneumonia.

## Figures and Tables

**Table 1:** CO-RADS categories and the corresponding level of suspicion for pulmonary involvement of COVID-19.

	Level of Suspicion for Pulmonary Involvement of COVID-19	Summary
CO-RADS 0	Not interpretable	Scan technically insufficient for assigning a score
CO-RADS 1	Very low	Normal or noninfectious
CO-RADS 2	Low	Typical for other infection but not COVID-19
CO-RADS 3	Indeterminate	Features compatible with COVID-19, but also other diseases
CO-RADS 4	High	Suspicious for COVID-19
CO-RADS 5	Very high	Typical COVID-19
CO-RADS 6	Proven	RT-PCR positive for SARS-CoV-2

CO-RADS, COVID-19 Reporting and Data System; COVID-19, coronavirus disease 2019; RT-PCR, reverse transcription-polymerase chain reaction; SARS-CoV-2, severe acute respiratory syndrome coronavirus 2.

**Table 2:** Characteristics of patients.

Parameter	COVID-19 Positive (76)	COVID-19 Negative (78)	All (144)	*p* COVID-19 Positive vs. Negative
Age (mean ± standard deviation (SD); range)	57.1 ± 19.3	65.4 ± 17.5	61.3 ± 18.8	0.006
	21–90	24–93	21–93	
Sex (*n*, % men)	54, 71.1%	47, 60.3%	101, 70.1%	0.215
Comorbidities (*n*, %)				
Cancer	9, 11.8%	30, 38.5%	39, 27.1%	<0.001
Cardiovascular disease	28, 36.8%	24, 30.8%	52, 36.1%	0.531
Lung disease	12, 15.8%	23, 29.5%	35, 24.3%	0.066
Collagen disease	5, 6.6%	16, 20.5%	21, 14.6%	0.022
Diabetes	10, 13.2%	7, 9.0%	17, 11.8%	0.568

COVID-19, coronavirus disease 2019; SD, standard deviation.

**Table 3:** List of diagnoses in COVID-19-negative patients.

Pneumonia Other than COVID-19	36
(Community-acquired pneumonia)	(29)
(Interstitial pneumonia)	(4)
(Drug-induced pneumonia)	(1)
(Eosinophilic pneumonia)	(1)
(Lung abscess)	(1)
Pulmonary edema	5
Atelectasis	4
Lung tumor	3
Pleuritis	2
Emphysema	1
Normal	27
*Total*	*78*

COVID-19, coronavirus disease 2019.

**Table 4:** Diagnostic performance and interobserver agreement of readers.

	Sensitivity	Specificity	AUC [95% CI]	ICC [95% CI]
Reader 1	80.3%	84.6%	0.847 [0.784–0.910]	0.800 [0.735–0.850]
Reader 2	90.8%	60.3%	0.849 [0.787–0.911]	0.874 [0.830–0.907]
Reader 3	85.9%	69.2%	0.904 [0.800–0.919]	0.855 [0.806–0.893]
Reader 4	93.4%	51.3%	0.881 [0.826–0.935]	0.829 [0.772–0.873]
Average of readers	87.8%	66.4%	0.859	0.840

Cut off value ≥ Category 3. AUC, area under the receiver operating characteristic curve; CI, confidence interval; ICC, intraclass correlation coefficient.

**Table 5:** Characteristics of false-positive cases in chest CT.

No	Sex	Age	CO-RADS Category (Median of 4 Readers)	Diagnoses
False-positive				(Final diagnoses)
1	Male	82	4	Community-acquired pneumonia
2	Male	59	4	Community-acquired pneumonia
3	Male	93	5	Community-acquired pneumonia
4	Male	73	4	Community-acquired pneumonia
5	Female	77	4	Community-acquired pneumonia
6	Male	77	4	Interstitial pneumonia
7	Female	54	4	Drug-induced pneumonia
8	Male	74	4	Eosinophilic pneumonia
9	Female	90	4	Pulmonary edema
10	Female	26	4	Pleuritis

CO-RADS, COVID-19 Reporting and Data System; CT, computed tomography.

**Table 6:** Characteristics of false-negative cases in chest CT.

No	Sex	Age	CO-RADS Category (Median of 4 Readers)	Onset to CT Duration (days)	Finding on Chest CT	Symptoms			
						Fever	Fatigue	Cough	Dyspnea
1	Male	25	2	9	Atelectasis	P	P	P	P
2	Female	24	1	19	Without abnormal findings	N	N	P	P
3	Female	25	1	11	Without abnormal findings	N	N	P	N
4	Female	30	1	26	Without abnormal findings	N	N	P	N
5	Male	90	1	5	Without abnormal findings	P	N	N	P
6	Female	25	1	25	Without abnormal findings	P	N	N	P

CO-RADS, COVID-19 Reporting and Data System; CT, computed tomography; P, positive; N, negative.

## References

[1] Zhu N., Zhang D., Wang W., Li X., Yang B., Song J., Zhao X., Huang B., Shi W., Lu R. (2020). A novel coronavirus from patients with pneumonia in China, 2019. N. Engl. J. Med..

[2] Novel Coronavirus (2019-nCoV) Situation Reports. https://www.who.int/emergencies/diseases/novel-coronavirus-2019/situation-reports.

[3] Riccò M., Ferraro P., Gualerzi G., Ranzieri S., Henry B.M., Said Y.B., Pyatigorskaya N.V., Nevolina E., Wu J., Bragazzi N.L. (2020). Point-of-care diagnostic tests for detecting SARS-CoV-2 antibodies: A systematic review and meta-analysis of real-world data. J. Clin. Med..

[4] Fang Y., Zhang H., Xie J., Lin M., Ying L., Pang P., Ji W. (2020). Sensitivity of chest CT for COVID-19: Comparison to RT-PCR. Radiology.

[5] Ai T., Yang Z., Hou H., Zhan C., Chen C., Lv W., Tao Q., Sun Z., Xia L. (2020). Correlation of chest CT and RT-PCR testing in coronavirus disease 2019 (COVID-19) in China: A report of 1014 cases. Radiology.

[6] Ojha V., Mani A., Pandey N.N., Sharma S., Kumar S. (2020). CT in coronavirus disease 2019 (COVID-19): A systematic review of chest CT findings in 4410 adult patients. Eur. Radiol..

[7] Sun Z., Zhang N., Li Y., Xu X. (2020). A systematic review of chest imaging findings in COVID-19. Quant. Imaging Med. Surg..

[8] Prokop M., van Everdingen W., van Rees Vellinga T., Quarles van Ufford J., Stöger L., Beenen L., Geurts B., Gietema H., Krdzalic J., Schaefer-Prokop C. (2020). CO-RADS-A categorical CT assessment scheme for patients with suspected COVID-19: Definition and. Radiology.

[9] Cellina M., Orsi M., Valenti Pittino C., Toluian T., Oliva G. (2020). Chest computed tomography findings of COVID-19 pneumonia: Pictorial essay with literature. Jpn. J. Radiol..

[10] Bulut C., Kato Y. (2020). Epidemiology of COVID-19. Turk. J. Med. Sci..

[11] Landis J.R., Koch G.G. (1977). The measurement of observer agreement for categorical data. Biometrics.

[12] Xiong F., Tang H., Liu L., Tu C., Tian J.B., Lei C.T., Liu J., Dong J.W., Chen W.L., Wang X.H. (2020). Clinical characteristics of and medical interventions for COVID-19 in hemodialysis patients in Wuhan, China. J. Am. Soc. Nephrol..

[13] Richardson S., Hirsch J.S., Narasimhan M., Crawford J.M., McGinn T., Davidson K.W., Barnaby D.P., Becker L.B., Chelico J.D., Cohen S.L. (2020). Presenting characteristics, comorbidities, and outcomes among 5700 patients hospitalized With COVID-19 in the New York city area. JAMA.

[14] Kim H., Hong H., Yoon S.H. (2020). Diagnostic performance of CT and reverse transcriptase-polymerase chain reaction for coronavirus disease 2019: A meta-analysis. Radiology.

[15] The Novel Coronavirus Pneumonia Emergency Response Epidemiology Team (2020). The epidemiological characteristics of an outbreak of 2019 novel coronavirus diseases (COVID-19)—China, 2020. China CDC Wkly..

[16] Fujioka T., Kubota K., Mori M., Kikuchi Y., Katsuta L., Kasahara M., Oda G., Ishiba T., Nakagawa T., Tateishi U. (2019). Distinction between benign and malignant breast masses at breast ultrasound using deep learning method with convolutional neural network. Jpn. J. Radiol..

[17] Fujioka T., Mori M., Kubota K., Kikuchi Y., Katsuta L., Adachi M., Oda G., Nakagawa T., Kitazume Y., Tateishi U. (2019). Breast ultrasound image synthesis using deep convolutional generative adversarial networks. Diagnostics.

[18] Adachi M., Fujioka T., Mori M., Kubota K., Kikuchi Y., Xiaotong W., Oyama J., Kimura K., Oda G., Nakagawa T. (2020). Detection and diagnosis of breast cancer using artificial intelligence based assessment of maximum intensity projection dynamic contrast-enhanced magnetic resonance images. Diagnostics.

[19] Fujioka T., Kubota K., Mori M., Kikuchi Y., Katsuta L., Kimura M., Yamaga E., Adachi M., Oda G., Nakagawa T. (2020). Efficient anomaly detection with generative adversarial network for breast ultrasound imaging. Diagnostics.

[20] Ni Q., Sun Z.Y., Qi L., Chen W., Yang Y., Wang L., Zhang X., Yang L., Fang Y., Xing Z. (2020). A deep learning approach to characterize 2019 coronavirus disease (COVID-19) pneumonia in chest CT images. Eur. Radiol..

[21] Li Y., Yang Z., Ai T., Wu S., Xia L. (2020). Association of “initial CT” findings with mortality in older patients with coronavirus disease 2019 (COVID-19). Eur. Radiol..

